# The impact of asthma, chronic bronchitis and allergic rhinitis on all-cause hospitalizations and limitations in daily activities: a population-based observational study

**DOI:** 10.1186/s12890-015-0008-0

**Published:** 2015-02-12

**Authors:** Simone Accordini, Angelo Guido Corsico, Lucia Calciano, Roberto Bono, Isa Cerveri, Alessandro Fois, Pietro Pirina, Roberta Tassinari, Giuseppe Verlato, Roberto de Marco

**Affiliations:** Unit of Epidemiology and Medical Statistics, Department of Public Health and Community Medicine, University of Verona, Verona, Italy; Division of Respiratory Diseases, IRCCS “San Matteo” Hospital Foundation, University of Pavia, Pavia, Italy; Department of Public Health and Pediatrics, University of Turin, Turin, Italy; Institute of Respiratory Diseases, University of Sassari, Sassari, Italy

**Keywords:** Burden of allergic rhinitis, Burden of asthma, Burden of chronic bronchitis, Determinants

## Abstract

**Background:**

Chronic respiratory diseases are a significant cause of morbidity and mortality worldwide. We sought to evaluate the impact of asthma, chronic bronchitis and allergic rhinitis on all-cause hospitalizations and limitations in daily activities in adults.

**Methods:**

In the Gene Environment Interactions in Respiratory Diseases study (2007/2010), a screening questionnaire was mailed to 9,739 subjects aged 20–44 (response rate: 53.0%) and to 3,480 subjects aged 45–64 (response rate: 62.3%), who were randomly selected from the general population in Italy. The questionnaire was used to: identify the responders who had asthma, chronic bronchitis, allergic rhinitis or asthma-like symptoms/dyspnoea/other nasal problems; evaluate the total burden [use of hospital services (at least one ED visit and/or one hospital admission) and number of days with reduced activities (lost working days and days with limited, not work related activities) due to any health problems (apart from accidents and injuries) in the past three months]; evaluate the contribution of breathing problems to the total burden (hospitalizations and number of days with reduced activities specifically due to breathing problems).

**Results:**

At any age, the all-cause hospitalization risk was about 6% among the subjects without any respiratory conditions, it increased to about 9-12% among the individuals with allergic rhinitis or with asthma-like symptoms/dyspnoea/other nasal problems, and it peaked at about 15-18% among the asthmatics with chronic bronchitis aged 20–44 and 45–64, respectively. The expected number of days with reduced activities due to any health problems increased from 1.5 among the subjects with no respiratory conditions in both the age classes, to 6.3 and 4.6 among the asthmatics with chronic bronchitis aged 20–44 and 45–64, respectively. The contribution of breathing problems to the total burden was the highest among the asthmatics with chronic bronchitis (23-29% of the hospitalization risk and 39-50% of the days with reduced activities, according to age).

**Conclusions:**

The impact of asthma, chronic bronchitis and allergic rhinitis on all-cause hospitalizations and limitations in daily activities is substantial, and it is markedly different among adults from the general population in Italy. The contribution of breathing problems to the total burden also varies according to the respiratory condition.

## Background

Asthma, chronic bronchitis and allergic rhinitis are common health problems in industrialized countries [1-3] and they often coexist [4-6]. Furthermore, chronic bronchitis is associated with both a more severe form of the disease and poor control of symptoms in subjects with asthma [7,8], and it may indicate the presence of the asthma-chronic obstructive pulmonary disease (COPD) overlap syndrome [9,10].

Asthma generates a high socio-economic burden among European adults, which significantly increases when chronic bronchitis is also present [11,12]. Moreover, asthma and allergic rhinitis together account for most of the allergy-related morbidity associated with the respiratory system [13]. Therefore, real-world evaluations of the impact of these diseases in the general population are fundamental to meet the widespread need for updated estimates, which should be used as a valid support to public health decisions.

In recent years, the socio-economic impact of asthma, chronic bronchitis and allergic rhinitis has been largely investigated [11,12,14-16]. However, in many studies, the total burden (i.e. the burden due to any health problems) in patients with these respiratory illnesses has not been estimated because only disease-related costs have been considered. Moreover, the total burden should be compared between patients with a certain disease and unaffected subjects from the general population. Indeed, a better understanding of the burden of these respiratory diseases requires the assessment of their total impact on the health and social systems.

The aim of the present paper is to compare all-cause hospitalizations and limitations in daily activities among adult subjects with asthma, chronic bronchitis or allergic rhinitis, and unaffected individuals from the general population, and to assess the contribution of breathing problems to the total burden. To fulfil these purposes, the data from the screening stage of the Gene Environment Interactions in Respiratory Diseases (GEIRD) study [17] were used.

## Methods

### Design of the study

The screening stage of GEIRD was a cross-sectional survey on respiratory health, which was carried out between 2007 and 2010 in Italy [17]. A total sample of 9,739 subjects aged 20–44 and of 3,480 subjects aged 45–64 (men/women ratio = 1) was randomly selected from the general population in four centres (Pavia, Sassari, Turin and Verona) by using the local health authority registers. A screening questionnaire was mailed to each individual up to three times and then administered by telephone in case of nonresponse. Overall, the responders were 5,162 (53.0%) in the 20–44 age group and 2,167 (62.3%) in the 45–64 age group (Table 1).

**Table 1:** **Distribution of the design variables**

		**20-44 yrs**	**45-64 yrs**	**p-value**
N° of eligible subjects*		9739	3480	-
N° of responders (response rate, %)		5162 (53.0)	2167 (62.3)	<0.001
Season at the time of response, %	Spring	46.2	51.5	<0.001
	Summer	15.2	8.6	
	Autumn	32.0	30.9	
	Winter	6.6	9.0	
Telephone interview, %		11.6	9.6	0.012
Females, %		53.6	52.1	0.267

*Eligible = initial sample – (dead or moved away from the target area).

### Ethics statement

Ethics approval was obtained in each centre from the appropriate ethics committee (“*Comitato di Bioetica della Fondazione IRCCS Policlinico San Matteo di Pavia*”; “*Comitato di Bioetica dell’Azienda Sanitaria Locale di Sassari*”; “*Comitato Etico dell’Azienda Sanitaria Locale TO/2 di Torino*”; “*Comitato Etico per la Sperimentazione dell'Azienda Ospedaliera Istituti Ospitalieri di Verona*”). All participants were fully informed about all aspects of the research project and consented to complete and return the questionnaire.

### Screening questionnaire and definitions

The screening questionnaire (available at www.geird.org) is a modified version of the questionnaire used in the Italian Study on Asthma in Young Adults [18]. It contains validated questions (mainly taken from the European Community Respiratory Health Survey questionnaires [19]) on asthma (self-report of the disease during lifetime with or without a physician’s diagnosis, frequency of asthma attacks, and use of anti-asthmatic drugs in the past 12 months), asthma-like symptoms (wheezing, nocturnal tightness in the chest, and attacks of shortness of breath at night time in the past 12 months), chronic cough and phlegm, and nasal problems, as well as questions on occupational status, smoking habits and outdoor air pollution (frequency of heavy traffic near home and living near industrial plants).

On the basis of their answers to the questions described above, the responders to the screening questionnaire were classified as subjects with:

- *asthma* (i.e. self-report of the disease during lifetime with or without a physician’s diagnosis or at least one asthma attack in the past 12 months or use of anti-asthmatic drugs in the past 12 months) *and chronic bronchitis* (i.e. cough and phlegm on most days for a minimum of three months a year and for at least two successive years);
- *chronic bronchitis without asthma*;
- *asthma without chronic bronchitis*;
- *allergic rhinitis* (i.e. any nasal allergies, including hay fever) *without asthma and chronic bronchitis*;
- *asthma-like symptoms or dyspnoea or other nasal problems without asthma, chronic bronchitis and allergic rhinitis*;
- *no respiratory conditions* (i.e. none of the conditions described above).

In addition, the responders were considered exposed to a high level of outdoor air pollution if they had reported a continuous passage of trucks near home and/or living near industrial plants.

The screening questionnaire also includes questions on the use of hospital services [at least one Emergency Department (ED) visit and/or one hospital admission], the number of lost working days and the number of days with limited, not work related activities (such as looking after children, housework or studying) due to any health problems (apart from accidents and injuries) in the past three months. If hospital services have been used, the responders should specify if this has been due to breathing problems. If lost working days and/or days with limited, not work related activities have been reported, the subjects should specify the number of the impaired days because of breathing problems.

The total burden was evaluated at an individual level on the basis of the use of hospital services and the number of days with reduced activities (i.e. the number of lost working days plus the number of days with limited, not work related activities) due to any health problems (apart from accidents and injuries) in the past three months. The days with limited, not work related activities reported by full-time house-persons were considered as lost working days. The hospital services utilization and the number of days with reduced activities specifically due to respiratory disorders in the past three months were used to evaluate the contribution of breathing problems to the total burden.

### Statistical analysis

The cumulative response rate, the season at the time of response to the questionnaire, the type of contact (postal waves vs telephone interview), and gender were considered as potential confounders because of differences in their distribution between the two age classes (20–44 and 45–64) (Table 1). In particular, the cumulative response percentile rank, which is an indicator of the individual propensity to respond to the questionnaire, was used. Each subject was ordered by centre and age class according to the date of response to the questionnaire, and then he/she was attributed the ratio between his/her rank and the number of eligible subjects [20].

The prevalence of each respiratory condition was estimated by using a logistic model, which included age class, centre and the potential confounders reported above, as covariates. The all-cause hospitalization risk [i.e. the proportion of subjects with at least one ED visit and/or one hospital admission due to any health problems (apart from accidents and injuries) in the past three months] and the expected number of days with reduced activities due to any health problems in the same period were estimated by using logistic and negative binomial models [21], respectively, which included the respiratory condition crossed by the age class, centre and the potential confounders reported above, as covariates. Since the subjects were nested into centres, clustered sandwich estimators of the variance were used. The estimates were computed in each age class and for each respiratory condition by setting the distribution of gender, season at the time of response to the questionnaire, type of contact and centre equal to the average distribution, and by setting the cumulative response rate equal to the overall mean [22].

A multivariable analysis was carried out to evaluate the association of each component of the total burden (i.e. all-cause hospitalizations and limitations in daily activities) with the respiratory condition, gender, occupational status, smoking habits and a high level of outdoor air pollution. Logistic and negative binomial models were computed separately in each age class and the estimates were adjusted for centre and the potential confounders reported above. Clustered sandwich estimators of the variance were used. The results of the negative binomial models were summarized as mutually adjusted Ratios of Expected Number of Days with reduced activities (RENDs).

In each age class and for each respiratory condition, the percentage contribution of breathing problems to all-cause hospitalizations and limitations in daily activities in the past three months was obtained by dividing the hospitalization risk and the expected number of days with reduced activities specifically due to respiratory disorders, by the corresponding component of the total burden. All the estimates of the hospitalization risk and of the expected number of days with reduced activities were adjusted for centre and the potential confounders reported above. Ninety-five percent confidence intervals (95%CIs) were computed by using the bias-corrected bootstrap method [23] with 2,000 replications.

The statistical analysis was performed by using STATA software, release 13 (StataCorp, College Station, TX).

## Results

### All-cause hospitalizations and limitations in daily activities according to the respiratory condition

**Figure 1:**
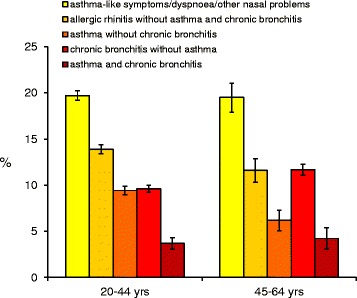
Prevalence* of each respiratory condition. Bars represent 95% confidence intervals. *The estimates were adjusted for the cumulative response rate, the season at the time of response to the questionnaire, the type of contact, gender and centre.

The percentage of subjects who reported any respiratory condition was 57.7% (95%CI: 57.4 to 58.0%) in the 20–44 age class and 54.8% (95%CI: 54.0 to 55.6%) in the 45–64 age class, with differences in the disease distribution according to age (Figure 1).

**Figure 2:**
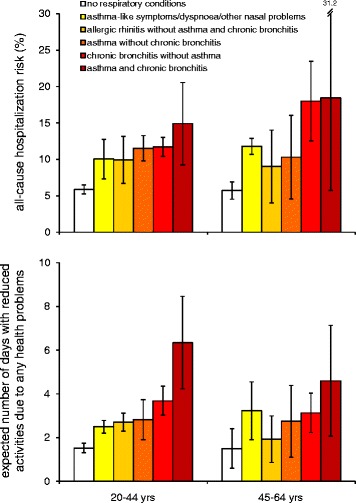
All-cause hospitalizations and limitations in daily activities in the past three months*. Bars represent 95% confidence intervals. *The all-cause hospitalization risk is the proportion of subjects with at least one ED visit and/or one hospital admission due to any health problems (apart from accidents and injuries) in the past three months; the estimates were adjusted for the cumulative response rate, the season at the time of response to the questionnaire, the type of contact, gender and centre.

The risk of having at least one ED visit and/or one hospital admission due to any health problems (apart from accidents and injuries) in the past three months was 8.8% (95%CI: 8.3 to 9.3%) and 9.5% (95%CI: 8.1 to 11.0%) among the subjects aged 20–44 and 45–64, respectively. At any age, the risk of all-cause hospitalization was about 6% among the subjects with no respiratory conditions, it increased to about 9-12% among the individuals reporting allergic rhinitis or asthma-like symptoms/dyspnoea/other nasal problems, and it peaked at about 15-18% among the asthmatics with chronic bronchitis aged 20–44 and 45–64, respectively (Figure 2). The same trend was observed after adjusting for the effect of the other potential determinants: the odds ratio (OR; reference category: no respiratory conditions) ranged from 1.62 to 2.36 in the 20–44 age class and from 2.09 to 4.24 in the 45–64 year-old group, respectively (Table 2).

**Table 2:** **Potential determinants of all-cause hospitalizations in the past three months**

		**20-44 yrs**		**45-64 yrs**	
		OR* [95% CI]	**p-value**	OR* [95% CI]	**p-value**
Respiratory condition	No respiratory conditions	1.00	-	1.00	-
	Asthma-like symptoms/dyspnoea/other nasal problems	1.62 [1.15 to 2.28]	0.005	2.09 [1.65 to 2.65]	<0.001
	Allergic rhinitis without asthma and chronic bronchitis	1.73 [1.09 to 2.73]	0.019	1.79 [1.07 to 2.98]	0.027
	Asthma without chronic bronchitis	2.08 [1.84 to 2.35]	<0.001	2.09 [1.32 to 3.32]	0.002
	Chronic bronchitis without asthma	1.85 [1.44 to 2.39]	<0.001	3.38 [1.95 to 5.88]	<0.001
	Asthma and chronic bronchitis	2.36 [1.45 to 3.84]	0.001	4.24 [2.06 to 8.70]	<0.001
Gender	Male	1.00	-	1.00	-
	Female	1.46 [1.04 to 2.07]	0.030	0.70 [0.53 to 0.91]	0.008
Occupational status	White collar/house-person/student	1.00	-	1.00	-
	Blue collar	1.31 [1.05 to 1.63]	0.016	0.74 [0.43 to 1.28]	0.278
	Unemployed/retired subject	1.60 [1.03 to 2.50]	0.038	1.39 [1.00 to 1.93]	0.049
Smoking habits	Never smoking	1.00	-	1.00	-
	Past smoking	1.22 [0.87 to 1.70]	0.252	1.20 [1.08 to 1.33]	0.001
	Current smoking	1.45 [0.96 to 2.18]	0.076	0.97 [0.62 to 1.53]	0.892
High level of outdoor air pollution	Absent	1.00	-	1.00	-
	Present	1.05 [0.81 to 1.37]	0.708	1.47 [1.18 to 1.84]	0.001

*Mutually adjusted odds ratios (ORs) of hospital services utilization (at least one ED visit and/or one hospital admission) due to any health problems (apart from accidents and injuries) in the past three months; the ORs were also adjusted for the cumulative response rate, the season at the time of response to the questionnaire, the type of contact and centre.

The expected number of days with reduced activities due to any health problems (apart from accidents and injuries) in the past three months was 2.4 (95%CI: 2.3 to 2.5) and 2.3 (95%CI: 2.1 to 2.5) among the subjects aged 20–44 and 45–64, respectively. This estimate increased from 1.5 days among the individuals with no respiratory conditions in both the age classes, to 6.3 and 4.6 days among the asthmatics with chronic bronchitis aged 20–44 and 45–64, respectively (Figure 2). The subjects aged 45–64 with asthma-like symptoms/dyspnoea/other nasal problems were characterised by a particularly high figure (3.2 days). The increasing trend in the number of days with reduced activities was confirmed after adjusting for the effect of the other potential determinants: the REND (reference category: no respiratory conditions) ranged from 1.64 to 3.87 in the 20–44 age class and from 2.05 to 4.33 in the 45–64 year-old group, respectively (Table 3).

**Table 3:** **Potential determinants of all-cause limitations in daily activities in the past three months**

		**20-44 yrs**		**45-64 yrs**	
		REND* [95% CI]	**p-value**	REND* [95% CI]	**p-value**
Respiratory condition	No respiratory conditions	1.00	-	1.00	-
	Asthma-like symptoms/dyspnoea/other nasal problems	1.64 [1.23 to 2.18]	0.001	2.05 [0.85 to 4.97]	0.112
	Allergic rhinitis without asthma and chronic bronchitis	1.72 [1.37 to 2.17]	<0.001	1.46 [0.64 to 3.31]	0.366
	Asthma without chronic bronchitis	1.76 [1.33 to 2.32]	<0.001	2.15 [1.43 to 3.22]	<0.001
	Chronic bronchitis without asthma	2.42 [1.90 to 3.08]	<0.001	3.19 [2.22 to 4.59]	<0.001
	Asthma and chronic bronchitis	3.87 [2.99 to 5.03]	<0.001	4.33 [2.10 to 8.89]	<0.001
Gender	Male	1.00	-	1.00	-
	Female	1.73 [1.37 to 2.18]	<0.001	0.95 [0.84 to 1.08]	0.436
Occupational status	White collar/house-person/student	1.00	-	1.00	-
	Blue collar	1.73 [1.57 to 1.91]	<0.001	2.17 [1.06 to 4.47]	0.035
	Unemployed/retired subject^†^	-	-	-	-
Smoking habits	Never smoking	1.00	-	1.00	-
	Past smoking	1.17 [0.97 to 1.40]	0.093	1.80 [1.22 to 2.66]	0.003
	Current smoking	1.07 [0.93 to 1.23]	0.324	0.66 [0.53 to 0.82]	<0.001
High level of outdoor air pollution	Absent	1.00	-	1.00	-
	Present	1.17 [0.88 to 1.55]	0.288	1.54 [0.94 to 2.54]	0.087

*Mutually adjusted ratios of expected number of days with reduced activities (RENDs) due to any health problems (apart from accidents and injuries) in the past three months; the RENDs were also adjusted for the cumulative response rate, the season at the time of response to the questionnaire, the type of contact and centre. ^†^The RENDs are not reported because unemployed/retired subjects have no lost working days.

### Contribution of breathing problems to all-cause hospitalizations and limitations in daily activities

**Figure 3:**
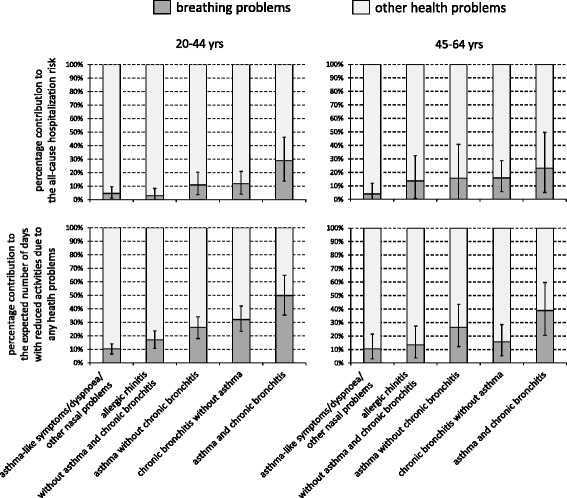
Percentage contribution of breathing problems to all-cause hospitalizations and limitations in daily activities in the past three months*. Bars represent 95% confidence intervals. *The all-cause hospitalization risk is the proportion of subjects with at least one ED visit and/or one hospital admission due to any health problems (apart from accidents and injuries) in the past three months; the estimates were adjusted for the cumulative response rate, the season at the time of response to the questionnaire, the type of contact, gender and centre.

Breathing problems accounted for less than 50% of all-cause hospitalizations and limitations in daily activities, and the percentage contribution of breathing problems to the total burden varied according to the respiratory condition in both the age classes (Figure 3). In particular, the highest contribution of breathing problems to the total burden was observed among the asthmatics with chronic bronchitis (23-29% of the hospitalization risk and 39-50% of the days with reduced activities, according to age), whereas the lowest figure was observed among the individuals with asthma-like symptoms/dyspnoea/other nasal problems (4-5% of the hospitalization risk and 10-11% of the days with reduced activities, in the two age groups).

### Other determinants of all-cause hospitalizations and limitations in daily activities

According to the multivariable analysis, females showed an increased hospitalization risk (OR: 1.46) and an increased number of days with reduced activities (REND: 1.73) in the population aged 20–44, whereas they had less hospitalizations (OR: 0.70) in the population aged 45–64, as compared to males in the same age class (Tables 2 and 3). As compared to white collars/house-persons/students of the same age, unemployed/retired subjects had an increased hospitalization risk in both the age classes (OR: 1.60 and 1.39, respectively), as found for blue collars (OR: 1.31) in the 20–44 age class; moreover, blue collars showed heavier limitations in daily activities in both the age groups (REND: 1.73 and 2.17, respectively). As compared to never smokers aged 45–64, past smokers had an increased hospitalization risk (OR: 1.20) and an increased number of days with reduced activities (REND: 1.80), whereas current smokers showed lesser limitations in daily activities (REND: 0.66). In the same age class, the individuals reporting a high level of outdoor air pollution showed an increased hospitalization risk (OR: 1.47), as compared to the subjects reporting a low exposure.

### Sensitivity analyses

The stability of the results was evaluated by repeating the analysis also adjusting for smoking habits or without adjusting for the cumulative response rate, the season at the time of response to the questionnaire and the type of contact. The estimates obtained under these two conditions were comparable with the figures computed in the main analysis (data not shown).

## Discussion

The main results of the present study are the following:

- the subjects with asthma, chronic bronchitis or allergic rhinitis have a two- to four-fold increased risk of all-cause hospitalizations and limitations in daily activities, as compared to unaffected individuals from the general population;
- among the subjects with asthma, chronic bronchitis or allergic rhinitis, breathing problems account for less than 50% of all-cause hospitalizations and limitations in daily activities, and the contribution of breathing problems to the total burden varies according to the respiratory condition;
- female gender, a low occupational status and a high level of outdoor air pollution contribute to the total burden.

### The presence of asthma, chronic bronchitis or allergic rhinitis is associated with an increase in all-cause hospitalizations and limitations in daily activities

Our data show that the risk of hospitalization and the number of days with impaired activities due to any health problems (apart from accidents and injuries) are impressively higher among adults with asthma, chronic bronchitis or allergic rhinitis, as compared to unaffected individuals from the general population in Italy. This result is in agreement with the findings in other studies, which show that respiratory symptoms are among the major causes of consultation at primary health care centres in different countries [24], and that these diseases impair work performance, social life and physical quality of life [25-27].

In each age class, the heaviest burden is associated with the coexistence of asthma and chronic bronchitis. In particular, the risk of all-cause hospitalization peaks among the 45–64 year-old subjects with both these conditions, whereas this risk is not so much higher than the figures observed among the individuals with the other respiratory conditions in the 20–44 age class. A possible explanation for this could be that the presence of chronic bronchitis may be an early expression of COPD in the older age group [28], whereas it may be an expression of post nasal drip among the individuals aged 20–44 [29].

Among the 45–64 year-old subjects, those reporting asthma-like symptoms/dyspnoea/other nasal problems show a heavy burden. This result could be explained by the age-related increase in the incidence of pneumonia, lung cancer, COPD or other diseases that cause dyspnoea or respiratory symptoms, which often require ED visits or hospital admissions.

### The contribution of breathing problems to all-cause hospitalizations and limitations in daily activities varies according to the respiratory condition

In our study, breathing problems account for less than 50% of all-cause hospitalizations and limitations in daily activities. This is not surprising because Druss and colleagues [30] reported that about one fourth of the costs due to five chronic conditions (mood disorders, diabetes, heart disease, asthma and hypertension) in the United States were incurred in treating the conditions themselves, whereas the remaining costs were due to coexisting illnesses.

The contribution of breathing problems to the total burden varies according to the respiratory condition, even if the results pertaining to the 45–64 age class should be interpreted with caution because of the large confidence intervals, which is due to the smaller sample size of that group, as compared with the dimension of the 20–44 age class. In particular, the highest weight of breathing problems is found among the subjects with coexisting asthma and chronic bronchitis, which suggests that the burden for these patients is mainly caused by the disease. This result indirectly confirms that chronic bronchitis is associated with both a more severe form of the disease and poor control of symptoms in asthmatic subjects [7,8], or it may be an expression of the coexistence of asthma and COPD [9,10]. Accordingly, poor control of symptoms and coexisting chronic bronchitis were both associated with increased disease-related costs in European adults with asthma [12]. On the contrary, the contribution of breathing problems is particularly low among the subjects with allergic rhinitis only, whereas it is at an intermediate level among those with asthma or chronic bronchitis only, which suggests a progressive increase in the level of impairment due to these respiratory diseases.

Breathing problems have the lowest weight in determining all-cause hospitalizations and limitations in daily activities for the subjects with asthma-like symptoms/dyspnoea/other nasal problems. Different factors may contribute to explain this finding. Smoking is associated with respiratory symptoms and it is strongly associated with cardiac and cerebrovascular diseases, and with diabetic complications. Dyspnoea and non-specific respiratory symptoms may be attributable to other illnesses, such as heart failure, essential hypertension, anaemia, electrolyte disorders or gastroesophageal reflux. Moreover, in the case of comorbidities such as diabetes, anaemia, hypertension or tachycardia, the decision to admit a patient can be based not only on factors that are directly related to the respiratory disease, but also on the difficulty in managing an outpatient. In fact, these patients are likely to require more health services and more complex health management strategies.

### Other determinants of all-cause hospitalizations and limitations in daily activities

Females aged 20–44 report a heavier burden as compared to males in the same age class, regardless of their disease status. In fact, females are probably more concerned about their health than males [31], as previously found among young adults with asthma in Italy [32]. On the contrary, the risk of all-cause hospitalization is lower among females aged 45–64, in accordance with official Italian statistics for the same period [33]. A heavier burden is reported by subjects with a low occupational status (i.e. blue collars and unemployed/retired individuals), in accordance with previous results for adult asthma in Italy [32]. This could be due to factors that affect health and are more frequent in lower social classes, such as residential and workplace pollutant exposures [34], or it could reflect a cumulative life course disadvantage [35]. Self-reported high levels of outdoor air pollution are associated with an increased risk of all-cause hospitalization among the subjects aged 45–64, which suggests that the exposure to air pollution has a long-term effect on health [36,37]. Finally, the pattern of association between smoking habits and the total burden could be due to the “healthy smoker effect” bias, since the subjects with the most severe underlying disease are the least likely to smoke [38].

### Strengths and weaknesses of the study

The main strength of the present analysis is that the data were collected through a highly standardized protocol [17], which ensures the comparability of the information among the centres. Moreover, the prevalence rates are based on self-reported symptoms, which were measured through an international validated questionnaire [18], and are less influenced by diagnostic procedures. In addition, the data were collected from patients who had been identified in the general population, rather than from clinically selected groups, which should guarantee that the studied sample encompasses a wide spectrum of respiratory conditions. Finally, a potential measurement error [39] could have influenced our estimates of the burden only to a minor extent, because our study did not involve elderly patients and the recall period considered in the questionnaire (three months) was short.

A few caveats should be taken into account when interpreting our results. Only four centres participating in GEIRD collected the information on the subjects aged 45–64 and so there could be a potential limitation in the generalizability of the results. Moreover, the participation rate was quite low (53.0%) among young adults, as observed in other epidemiological studies over the past decades [40], and this may have biased our estimates for this age class. However, it has been suggested that decreasing participation rates are not likely to have a substantial influence on the point estimates of measures of interest [40]. Finally, it was not possible to directly evaluate the impact of comorbidities on the total burden because questions on coexisting diseases had not been included in the screening questionnaire, in order to minimize its length and therefore increase the response rate [18]. In addition, the screening questionnaire does not allow to compute separate estimates of the risk of having at least one ED visit and of the risk of having at least one hospital admission in the past three months. Therefore, the possible overestimation of hospital services utilization cannot be detected through the comparison with ED visit rates and hospital in-patient admission rates from other sources. However, participation bias could have inflated our estimates of the hospitalization risk to some extent.

## Conclusions

The impact of asthma, chronic bronchitis and allergic rhinitis on all-cause hospitalizations and limitations in daily activities is substantial, and it is markedly different among adult subjects from the general population in Italy. The contribution of breathing problems to the total burden also varies according to the respiratory condition. Gender, occupational status and outdoor air pollution contribute to the total burden.

## Abbreviations

COPD: Chronic obstructive pulmonary disease
GEIRD: Gene Environment Interactions in Respiratory Diseases
ED: Emergency Department
RENDs: Ratios of Expected Number of Days with reduced activities
95%CI: 95% confidence interval
OR: Odds ratio
